# NGS implementation for monitoring SARS-CoV-2 variants in Chicagoland: An institutional perspective, successes and challenges

**DOI:** 10.3389/fpubh.2023.1177695

**Published:** 2023-04-20

**Authors:** Aileen C. Tartanian, Nicole Mulroney, Kelly Poselenzny, Michael Akroush, Trevor Unger, Donald L. Helseth, Linda M. Sabatini, Michael Bouma, Paige M.K. Larkin

**Affiliations:** ^1^NorthShore University HealthSystem, Evanston, IL, United States; ^2^Pritzker School of Medicine, The University of Chicago, Chicago, IL, United States

**Keywords:** SARS-CoV-2, sequencing, molecular microbiology, molecular diagnostics, next generating sequencing

## Abstract

Identification of SARS-CoV-2 lineages has shown to provide invaluable information regarding treatment efficacy, viral transmissibility, disease severity, and immune evasion. These benefits provide institutions with an expectation of high informational upside with little insight in regards to practicality with implementation and execution of such high complexity testing in the midst of a pandemic. This article details our institution’s experience implementing and using Next Generation Sequencing (NGS) to monitor SARS-CoV-2 lineages in the northern Chicagoland area throughout the pandemic. To date, we have sequenced nearly 7,000 previously known SARS-CoV-2 positive samples from various patient populations (e.g., outpatient, inpatient, and outreach sites) to reduce bias in sampling. As a result, our hospital was guided while making crucial decisions about staffing, masking, and other infection control measures during the pandemic. While beneficial, establishing this NGS procedure was challenging, with countless considerations at every stage of assay development and validation. Reduced staffing prompted transition from a manual to automated high throughput workflow, requiring further validation, lab space, and instrumentation. Data management and IT security were additional considerations that delayed implementation and dictated our bioinformatic capabilities. Taken together, our experience highlights the obstacles and triumphs of SARS-CoV-2 sequencing.

## Introduction

Next generation sequencing (NGS) has been pivotal for understanding the impact of SARS-CoV-2 variants on transmission, pathogenicity, disease severity, vaccine and therapy efficacy, and diagnostic detection (1). For instance, the Omicron variant has been shown to evade the immune response in patients previously infected with SARS-CoV-2 or vaccinated against SARS-CoV-2 (2–5), render the majority of monoclonal antibody therapies ineffective (4), and cause more infections in younger patients compared to other variants (6). Conversely, the Omicron variant was associated with a lower 28-day mortality, ICU admission rate, and oxygen requirements compared to Delta (7).

With the benefits of SARS-CoV-2 sequencing highlighted, implementing such testing is attractive to many healthcare systems, but there are numerous challenges and considerations that should be addressed. Here we describe our experience with implementing NGS for SARS-CoV-2 variant analysis at NorthShore University HealthSystem (NSUHS) molecular diagnostic laboratory (MDL). As a fully integrated healthcare system, NSUHS-Edward-Elmhurst Health (EEH) serves over 4.2 million residents across northeast Illinois, including the city of Chicago and six suburban counties. The system currently encompasses 8 hospitals and over 300 outpatient centers. The NSUHS MDL was the first clinical laboratory in Illinois to perform SARS-CoV-2 testing (8) and has performed over 800,000 SARS-CoV-2 diagnostic assays to date. Our initial goals of SARS-CoV-2 sequencing was to detect shifts and emergence of lineages in real time, but challenges with staffing, turn-around-time (TAT), and sample selection complicated this.

## Demand on the lab and lab staff

The MDL has ample experience with NGS, given the breadth of oncology NGS assays performed, uniquely positioning the laboratory to bring in SARS-CoV-2 sequencing compared to laboratories without sequencing experience. We officially launched COVIDSeq on an Illumina NextSeq 550Dx (San Diego, CA, United States) in March of 2021 after delays due to installation, training, and reagent acquisition. Once launched, COVIDSeq productivity was constrained by the priority given to clinical diagnostic assays for staffing and freezer storage. Based on these factors, the percentage of SARS-CoV-2 samples tested by the MDL that progressed to sequencing ranged from 0 to 18.5% monthly for 2022, when sequencing was performed on a regular basis (Table 1). Samples were selected based on testing location and available media volume, with the exact number of samples tested fluctuating due to balancing the cost of a run and the availability of reagents and technologists to perform sequencing within a timeframe. Samples from 2020 and 2021 were run retrospectively, but due to delays discussed in detail below, most of 2021 was spent troubleshooting, validating, and optimizing. The use of a manual bioinformatics pipeline and analysis, as discussed below, complicated analysis. The addition of a new technologist to lead SARS-CoV-2 sequencing and move to more automation, both for the wet lab and dry lab components, facilitated more streamlined SARS-CoV-2 sequencing in 2022. Looking at 2022, there were factors that directly impacted the number of samples that could be sequenced per month. In September, we extracted and prepared libraries for 163 samples. However, our liquid handler malfunctioned by erroneously releasing all pipette tips and crashing the program. All samples had been depleted and the libraries were rendered unsavable. In November and December 2022, we exhausted our purchased sequencing reagents and did not have approval to order additional sequencing reagents due to the high cost that exceeded the allotted budget. All SARS-CoV-2 sequencing was self-funded by our institution, requiring careful planning and restricting the ability to expand sequencing capacity significantly.

**Table 1:** Summary of SARS-CoV-2 samples tested clinically and sequenced at NSUHS for 2022.

Month (2022)	# SARS-CoV-2 clinically tested	# Positive SARS-CoV-2	# Positives sequenced	# Samples of sequenced with consensus sequence	% Samples of positives sequenced	% Samples of sequenced positives with a consensus sequence
January	25,892	6,062	607	272	10.0	44.8
February	20,396	1,523	281	71	18.5	25.3
March	24,870	1,133	134	35	11.8	26.1
April	30,301	3,065	258	147	8.4	57.0
May	33,106	5,963	432	211	7.2	48.8
June	24,988	4,423	433	170	9.8	39.3
July	23,230	4,556	482	252	10.6	52.3
August	22,467	3,674	299	119	8.1	39.8
September	22,467	2,461	0	N/A	0.0	N/A
October	24,972	2,394	297	75	12.4	25.3
November	28,592	2,908	0	N/A	0.0	N/A
December	27,763	3,857	0	N/A	0.0	N/A

Our health system utilizes several different RT-PCR platforms for SARS-CoV-2 testing, which supports large volume testing in a variety of settings, including point-of-care and at each of our hospitals. However, this also led to multiple different swabs, transport media, and sample volumes. These variations were due to different assay requirements, sporadic swab and transport media shortages, and testing locations stocking different swabs. Due to early implementation of SARS-CoV-2 RT-PCR testing, we performed testing for a number of outreach non-affiliated sites that used a variety of swabs. The utilization of multiple instruments, many without available cycle threshold (Ct) values prevented establishment and selection of samples with appropriate Ct values. Often, labs will set a minimum Ct value for sequencing to increase sequencing yield, but we did not have that ability given the lack of available Ct value data. With sequencing any positive sample in 2022, only 25.3–57.0% of positive samples resulted in a consensus sequence for a SARS-CoV-2 lineage (Table 1). Some explanations for this wide range include low viral load (most EUA platforms used for clinical testing at our healthcare system only provided positive/negative results without a Ct value), and user and instrument error, including issues described below when automation was implemented. Due to lack of staffing and available resources, we did not collect this data for 2021 as we had to retrospectively sequence samples.

## Complications with manual processing

The lab performed SARS-CoV-2 library preparations and sequencing using Illumina’s COVIDSeq^™^ RUO Test. As with most NGS assays, the library preparation portion of COVIDSeq is costly in terms of time and labor. Initially, all library preparations were performed manually, requiring two full 8 h shifts for one technologist to complete (Table 2). While there were two technologists trained on the COVIDSeq assay, these technologists also were performing molecular diagnostic assays for clinical care, limiting their ability to prepare libraries for COVIDSeq to once per week.

**Table 2:** Comparison of manual vs. automated library preparations.

	Manual (96 samples)	Automated (48 samples)
No. batches per sequencing run	4	8
No. of pipette tip boxes	33	5
Hands-on tech time	16 h	3 h→30 min
Turn around time	2 days	7 h
Final pool molarity	>150 nM	<8 nM

The increased demand for clinical plastic consumables caused backorders and supply chain issues, restricting our ability to regularly perform COVIDSeq. During the first year, it was difficult to acquire an adequate amount of pipette tips to perform not only COVIDSeq but any of our routine molecular diagnostic clinical assays. Manually performing one COVIDSeq library preparation would consume 33 pipette tip boxes (Table 2). Therefore, to sequence a full run of 384 samples, over 130 tip boxes would be required. Regular and rapid sequencing during shifts and emergence of lineages such as Alpha, Delta, and Omicron would have been beneficial as these results would contribute to the local and global sequencing effort as well as guide hospital policies (e.g., allowed meeting size). However, the clinical assays consumed the necessary pipette tips and other plastic consumables so this was not feasible.

## Complications with automatic processing

With technologist time and consumables preciously scarce, two automated liquid handlers were purchased to supplement the labor demand required for this initiative. The PerkinElmer Janus G3 liquid handler (Waltham, MA, United States) was chosen to facilitate RNA extraction because it had a high volume capacity and was relatively easy to use. Unfortunately, several calibration corrections were required after initial install due to persistent issues with probe pressure and pipette tip compatibility resulting in inconsistent reagent and sample volumes. Most calibrations would require the onsite visit of a field service technician, delaying implementation even further. Once resolved, Janus was compatible with our already implemented ThermoFisher KingFisher Flex (Waltham, MA, United States) instrument for viral RNA extraction/purification.

The Beckman Coulter i7 liquid handler (Brea, CA, United States) was purchased to automate library preparation. Because COVIDSeq library preparations of 96 samples require two thermocyclers running in tandem, and the i7 had only one, the batches were halved from 96 to 48 to accommodate the missing thermocycler. The i7 reduced hands-on time from 2 days to 7 h. In addition, the i7 uses only 30% of the number of tip boxes (Table 2). While there was a greater supply of tips for the i7 compared to manual pipette tips, we went one step further to decrease our chances of competing with labs for tips by using the unusual pipette tip size of 190 μL.

The i7 is convenient and improves workflow, but there were many challenges in establishing this assay automation. For instance, hard shell 96-well plates were on backorder when the i7 arrived, so we used non-hardshell 96-well plates. These plates melted and warped from the heat of the thermocycler, causing the i7 to drop, crush, and toss the plates as the grippers attempted to move them. Similarly, these plates proved to be incompatible with the reusable lid used by the i7 thermocycler. During a run, the i7 would sense every time the plate was improperly sealed and stop the program. These problems required constant attention by our lab staff, manually adjusting the fit of the thermocycler lid. This persisted until the correct 96-well plates could be obtained.

Automated liquid handlers can be the source of numerous errors that are difficult to identify and troubleshoot. For example, we observed a consistent reduction in consensus sequence yield for samples positioned on the left side of the 96-well plates compared to the right side. After troubleshooting, the lab determined that the i7 instrument lacked steps within the run script to re-suspend magnetic beads prior to arraying them into samples. As the beads settled to the bottom of the source tube through the duration of the library preparation, the i7 would dispense bead storage buffer, absent of beads, to the samples on the left side of the plate while the samples on the right side received the majority of beads. Once the mixing step was supplemented into the run script, we noted an improved uniformity of sample performance coupled with vastly improved library concentration yield. Despite this fix, the library concentration from the i7 would remain inferior to the yield of manual library preparation. And, like the Janus, i7 calibrations, updates, and repairs would often be delayed because they required an onsite visit from a service technician.

We continued to identify opportunities for improved efficiency. The COVIDSeq program on our i7 calls for all reagents to be placed in 1.5 ml tubes and kept chilled on a cold block on the deck, reducing hands-on work for technologists, but it could take up to 3 h for a technologist to prepare the 1.5 ml tubes of master mixes. Over time, we reduced this timeframe by over 80% because we found that many of these master mixes could be prepared and frozen in advance without sacrificing library preparation performance (Table 2).

Manual library preparations would typically produce >150 nM pooled libraries, but switching to automation resulted in libraries of <8 nM, despite the corrections made to the run script. Pools with a molarity <0.5 nM would result in a total batch failure defined as a 0% consensus sequence. However, above 0.5 nM, we found no correlation (*R*^2^ = 0.0442) between a pool’s molarity and the percentage of samples resulting in a consensus sequence (Supplementary Figure S1). Ideally, we would quantify all individual specimens after RNA extraction, cDNA synthesis, and library preparation, removing low-concentration samples at each QC step. However, our lab does not have a high throughput way to quantify 96 samples at a time, so we only quantified each batch’s pooled library prior to combining the pools for sequencing.

## Bioinformatics and cybersecurity

NGS generates millions of sequencing reads per sample, and analyzing these reads requires a robust bioinformatics pipeline in an effort to detect and track novel variants. When the bioinformatics infrastructure is insufficient to support this immense quantity of data, institutions typically opt for commercially available solutions; either cloud-based or local, for their bioinformatics pipeline needs due to ease of use and readily available customer support. Cloud-based applications have the benefit of ease-of-use and easily accessible vendor support; however, the ever-growing push for cloud application usage provides tremendous cybersecurity concern for institutions and often requires a lengthy and in-depth risk assessment, which can delay implementation. Using a local analysis bioinformatics application platform can reduce concern from a cybersecurity perspective, but it increases cost as these systems often require the purchase of licensed software and additional hardware.

Our initiative to implement a SARS-CoV-2 NGS assay was driven by immediate need to contribute in variant tracking within our community. Due to urgent importance and to avoid further delay in implementation, we opted to purchase the local Illumina DRAGEN server (as opposed to Illumina’s cloud-based application BaseSpace) to be the primary source of our bioinformatics data analysis. At the time, a BaseSpace subscription would have forced an extensive, lengthy risk assessment by our cybersecurity team as these cloud-based applications do not always satisfy standard HIPAA requirements to protect personal health information (PHI).

The DRAGEN COVIDSeq test local pipeline provided a summary report of positive or negative results along with output directories containing the desired FASTA and VCF files. FASTAs, BAMs and VCFs generated by the Illumina DRAGEN software on the NextSeq 550Dx sequencer were copied to a separate Linux server for analysis. Initially, we ran our own variant calling pipeline using open source software (using samtools), visualizing the results in IGV, and running a local copy of Ensembl VEP for COVID-19 to annotate the variant consequences. This labor-intensive effort was quickly abandoned when we began using more specialized open source software packages provided by Nextstrain, Pangolin, and Nextclade, reducing the necessity of manual analysis. After using Nextstrain (9) for a few months, we recognized that variant nomenclature was evolving away from Nextstrain clade names to Pango lineage (10) and WHO labels. To generate Pango names we analyzed merged FASTA files using the latest version of Pangolin (11). Nextclade (12) was also used to compare and summarize variant classifications by uploading our merged FASTA files (13).

Launching the DRAGEN COVIDSeq local pipeline was initiated via the Linux command line terminal. This method is extremely foreign to users who are accustomed to GUI-based software with only little to moderate Linux command line experience. Customer support was a necessity, particularly support via vendor remote access, as we experienced frequent pipeline analysis failures along with connectivity issues between the DRAGEN server and the NextSeq 550Dx. Vendor bioinformatics support is generally equipped to support their customers remotely. NorthShore HIT did not permit vendor remote support access, limiting our only options to lengthy phone conversations or email correspondence. With restricted remote access to independently investigate and troubleshoot, vendors rely on these often mutually time consuming methods to investigate and eventually resolve the issue. Lack of proper vendor remote support to address these issues contributed to lengthy delays in data processing as resolution to these problems often extended across multiple days.

With this workflow, we quickly realized that our goal to track lineage shifts in real time would be extremely difficult to accomplish. Available bandwidth for our highly talented yet small bioinformatics team was limited, as our established clinical oncology NGS assays were beginning to rebound to pre-pandemic volumes. Building and maintaining a local pipeline intended to track current lineages shifts required a considerable amount of bioinformatics support beyond the limits of our available institutional resources.

Internal bioinformatics resources were not the only struggle experienced through this initial process. The laboratory workflow required for the DRAGEN COVIDSeq test pipeline included a requirement for a positive, negative, and no template control for each set of 96 indices to be included in each sequencing run. In the event of a control failure, the entire set of 96 samples became invalid. To avoid risk of control failure, each positive control required a fresh serial dilution prior to each library preparation. These dilutions were not recommended to be stored long term. Since our intent was to only sequence known SARS-CoV-2 positive samples, the inclusion of controls seemed to hold little value and only added complexity to the workflow.

The local DRAGEN COVIDSeq pipeline did provide some upside. Each analysis completed rather quickly (usually within 1 to 2 h) and provided the necessary output data required for lineage identification. However, because the workflow to maintain this pipeline became unmanageable, we made the decision to purchase a BaseSpace subscription and shift our analysis to this cloud-based application. This transition required a lengthy approval process through our HIT cybersecurity team as cloud-based NGS data analysis increases potential risk to loss of PHI. To diminish this risk, we decided that all samples would remain de-identified throughout the wet bench, sequencing, and post sequencing analysis. All data would be presented as aggregated de-identified data with no link to clinical information. We did not have permission from HIT to submit any data to GISAID. Not only were our samples de-identified on the sequencer, but our institution considers date collected as PHI. This information is requested by GISAID for submission. Clinical microbiology laboratories at other institutions were able to submit completely anonymized samples to their academic colleagues for sequencing and in turn were able to successfully report de-identified metadata to GISAID and NCBI (14). These labs had IRBs that allowed patient-level data to be reported back to public health entities as the clinical labs retained access to the patient-level data while the academic sequencing partners did not have access (14). This approach, which requires institutional approval, infrastructure for de-identifying and re-identifying, and access to academic sequencing laboratories, would be ideal to allow dissemination of data to public health and biorepositories. In our case, sequencing was so delayed that our public health colleagues would have already sequenced those samples of interest, creating another hurdle for rapid collaboration.

Using the DRAGEN COVIDSeq pipeline via BaseSpace Sequence Hub resolved many of the previously mentioned concerns, including ease of use. Although analysis times increased by four-fold due to the shared traffic of the cloud-based server, the data analysis process was exponentially easier as it required very little intervention from internal staff and remote support was easily available to resolve problems. However, when launching the DRAGEN COVID lineage application, the sample selection process seems to be the most taxing step. Samples can be selected in groups, but careful attention is required as it is easy to unintentionally include or exclude samples from analysis. Identifying samples to be analyzed through the application can be difficult as the sample list includes both completed and analysis-pending samples. These concerns are rather minor compared to our prior workflow and the DRAGEN COVID lineage application has provided a manageable data analysis workflow as the application provides mapping/alignment and variant calling features. Open source databases, like NextClade and Pangolin, are routinely updated and made available for analysis through the application, and the data is easily viewable and managed by multiple users.

## Clinical relevance/discussion

Molecular diagnostic assays that directly impact patient care were prioritized over SARS-CoV-2 sequencing, posing a challenge to continue RUO sequencing at high capacity. This was particularly the case during SARS-CoV-2 waves, when staffing was reduced due to illness and supplies were in high demand (15). As a result, there were substantial delays (>1 month) in sequencing SARS-CoV-2 specimens, contrasting with our original plan of using COVIDSeq to capture shifts and emergence of SARS-CoV-2 lineages. In addition to the cost, sequencing all specimens would likely provide little additional information as most samples received during pandemic waves would have the same composition of lineages that would be better captured with a smaller representative sample selection. On the other hand, between waves, our sample volume was too low to form any statistically relevant conclusions. Furthermore, it would take substantial time to accumulate 384 specimens for a full sequencing run, delaying results or forcing a partial run, which was costly. Surges in cases led us to recruit additional resource staff and research lab team members to work additional shifts to propel sequencing efforts, manually sorting through the samples to confirm positives, creating specimen labels, aliquoting, and documenting.

As previously discussed, our results were de-identified and mass aggregated to demonstrates shifts and trends within our patient population. While ideally we could share our results with our local health department to aid in their sequencing efforts, our results were not only delayed, but also did not have linked clinical data. This meant that sequencing efforts were unnecessarily duplicated due to inability to coordinate and share results, furthering the documented gap between public health labs and clinical labs (1, 16). We were, however, able to capture data categorized by symptomatic vs. asymptomatic cases and had these samples designated with their own test code for easy sorting and comparison. This comparison relied on trusting that physicians selected the correct test code indicating the presence or absence of symptoms. While we had planned to use these data to make comparisons between lineage and symptomatic state, upon review, we found that a small portion of physicians erroneously ordered the wrong test code and thus, accurate conclusions required substantial review. If the test codes had been appropriately ordered, the comparison in lineage between symptomatic and asymptomatic patients could have contributed to our knowledge in the field. In the end, we were able to share monthly trends with our healthcare system, modeling what other institutions have done (14).

Our decisions to sequence various populations and ultimately switch to mostly inpatient and ED specimens likely resulted in selection bias toward patients whose SARS-CoV-2 infection was not only symptomatic, but severe enough to seek hospital treatment, as well as selecting toward patients from high risk ages (including infants and those over the age of 65 years old) and individuals with pre-existing health conditions. The challenges of inferring clinical impact of variants have been well documented (17) as it is impossible to get a truly representative sample. Severity of symptoms is subjective and testing restrictions fluctuated throughout the pandemic, with some hospital systems only allowing the sickest patients to get tested (17). Moreover, COVID-19 studies often focus on hospitalized patients, not representative of the general population (17). The shift to at-home antigen testing also biases against sequencing asymptomatic or mildly symptomatic patients (16).

Despite the issues discussed previously, our data were useful in a broader capacity for our healthcare system. While there were detected cases of Omicron in our state, our sequencing confirmed the presence of Omicron in our patient population. This contributed to discussions on policies for masking and permitted meeting sizes. Furthermore, in conjunction with *in silico* analysis, we used COVIDSeq to test detection of sequence-confirmed variants in our lab-developed SARS-CoV-2 assay. We were able to confirm that the primers for this clinical assay could still detect even the most recently detected lineages of SARS-CoV-2. This was a concern for clinical laboratories across the world as the Alpha and Omicron variants exhibited spike (S) gene dropouts on assays that detect the S gene (18). While we do not currently utilize any assay that targets S gene, mutations can occur in any region of the genome and thus it is important to monitor whether these mutations impact the ability for our assays to detect SARS-CoV-2.

The question of balancing cost, in terms of time and money, as well as staffing remains difficult and potentially unsustainable in the long-term for genomic surveillance. At times where multiple lineages are circulating, there was a push for more sequencing to better document lineage changes within our patient population, with the caveat that we do not have the capacity to provide rapid TAT for COVIDSeq. When there was an overwhelmingly predominant lineage, there was less institutional support for routine sequencing as, until a new variant of interest or concern is identified or mutations within current circulating variants would render treatments ineffective, the results would not impact hospital protocols. However, this approach would prevent detection of shifts in lineages as well as detection of novel lineages.

The challenges described here were not unique to our health care system. Both the importance of localized surveillance efforts as well as the extensive challenges in terms of labor force, supply chain issues, and coordinated data acquisition, analysis and sharing became painfully evident. In recognition, Congress passed the “Tracking Pathogen Act” as part of pandemic preparedness measures within the FY2023 Omnibus legislation. This Act directs the Department of Health and Human Services to issue guidance and to support such efforts.

## Data availability statement

The raw data supporting the conclusions of this article will be made available by the authors, without undue reservation.

## Author contributions

AT: writing—original draft, writing—review and editing, visualization, data curation, and investigation. NM, KP, MA, and TU: writing—review and editing, data curation, and investigation. DH: writing—review and editing, formal analysis, and software. LS: writing—review and editing, supervision, and conceptualization. MB: writing—conceptualization, original draft, writing—review and editing, supervision, data curation, and formal analysis. PL: writing—conceptualization, original draft, writing—review and editing, and project administration. All authors contributed to the article and approved the submitted version.

## Conflict of interest

The authors declare that the research was conducted in the absence of any commercial or financial relationships that could be construed as a potential conflict of interest.

## Publisher’s note

All claims expressed in this article are solely those of the authors and do not necessarily represent those of their affiliated organizations, or those of the publisher, the editors and the reviewers. Any product that may be evaluated in this article, or claim that may be made by its manufacturer, is not guaranteed or endorsed by the publisher.

## Supplementary material

Supplementary Figure 1 Lack of Correlation Between Library Pool Molarity and Percentage of Samples Generating a Consensus Sequence.
