# Corrigendum: NGS implementation for monitoring SARS-CoV-2 variants in Chicagoland: an institutional perspective, successes and challenges

**DOI:** 10.3389/fpubh.2023.1234900

**Published:** 2023-06-27

**Authors:** Aileen C. Tartanian, Nicole Mulroney, Kelly Poselenzny, Michael Akroush, Trevor Unger, Donald L. Helseth, Linda M. Sabatini, Michael Bouma, Paige M. K. Larkin

**Affiliations:** ^1^NorthShore University HealthSystem, Evanston, IL, United States; ^2^Pritzker School of Medicine, The University of Chicago, Chicago, IL, United States

**Keywords:** SARS-CoV-2, sequencing, molecular microbiology, molecular diagnostics, next generating sequencing

In the published article, an author name was incorrectly written as Aileen C. Tartaninan. The correct spelling is Aileen C. Tartanian.

The authors apologize for this error and state that this does not change the scientific conclusions of the article in any way. The original article has been updated.

