# Supplementary figures and images for: NGS implementation for monitoring SARS-CoV-2 variants in Chicagoland: An institutional perspective, successes and challenges
Source: Front Public Health. 2023 Apr 20;11:1177695. doi: 10.3389/fpubh.2023.1177695 (PMC10157391; doi:10.3389/fpubh.2023.1177695)

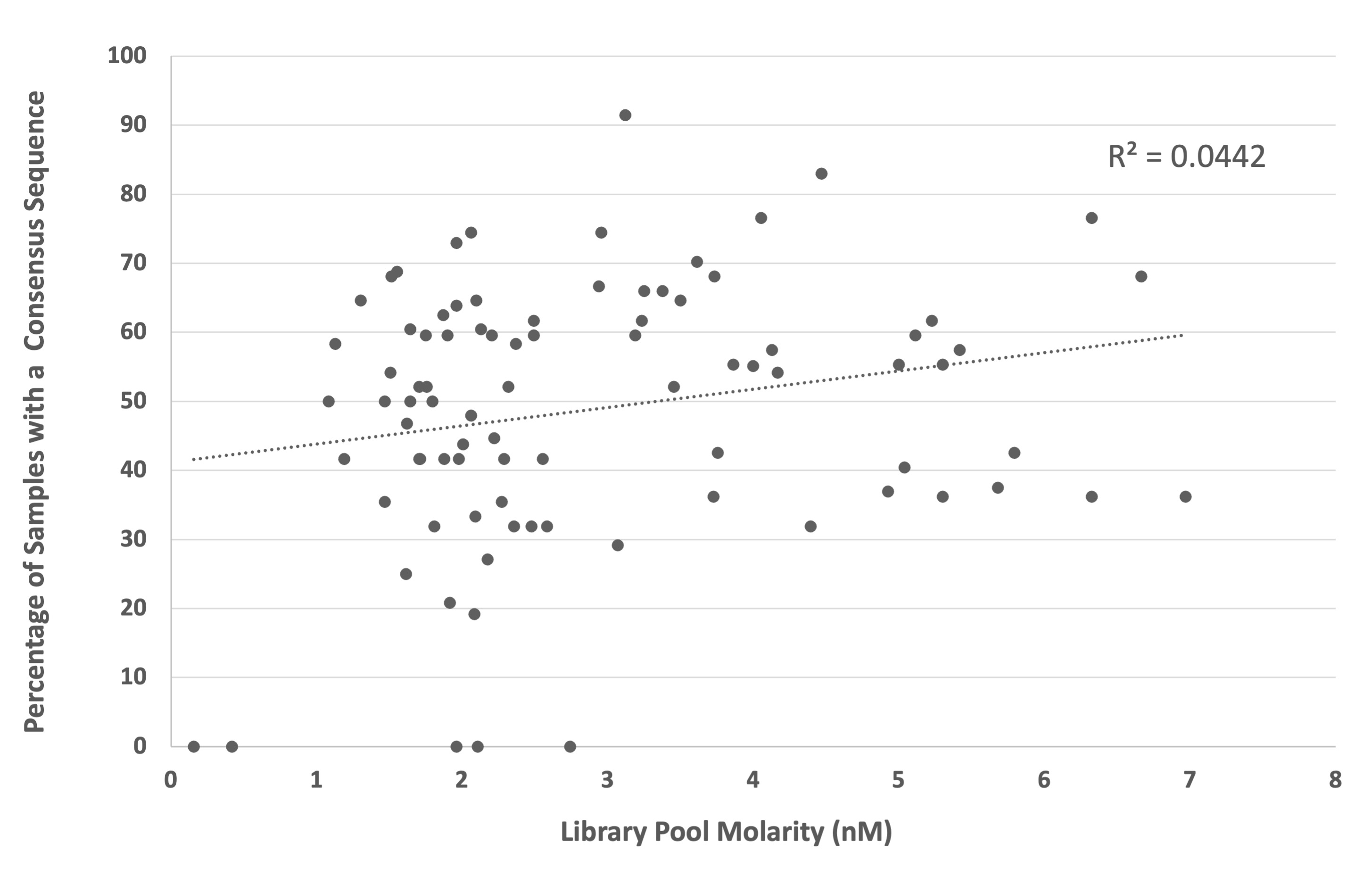

Supplement: Supplementary Figure 1 — Lack of Correlation Between Library Pool Molarity and Percentage of Samples Generating a Consensus Sequence. [file Image_1.JPEG]
